# Agonists and Antagonists Show Different Unbinding Paths from the TLR8 Receptor

**DOI:** 10.1021/acs.jcim.5c00496

**Published:** 2025-07-09

**Authors:** Valerij Talagayev, Gerhard Wolber, Ariane Nunes-Alves

**Affiliations:** † Department of Pharmaceutical and Medicinal Chemistry, Institute of Pharmacy, 9166 Freie Universität Berlin, Königin-Luise-Str. 2+4, Berlin 14195, Germany; ‡ Institute of Chemistry, 26524 Technische Universität Berlin, Straße des 17. Juni 135, Berlin 10623, Germany

## Abstract

Toll-like receptors (TLRs) form the first barrier of the innate immune system. TLR8 is an important target to treat autoimmune diseases since its ligand-induced degree of activation regulates immune response and associated hyperinflammation. Molecular dynamics (MD) simulations have been used to investigate interactions of TLRs with ligands, but the mechanism of ligand unbinding remains elusive. We therefore applied τ-random acceleration molecular dynamics (τRAMD) simulations to characterize the unbinding paths of one TLR8 agonist and five TLR8 antagonists. Data analysis of the simulations led to the discovery of two possible unbinding pathways: the internal pathway, directed toward the Toll-interleukin-1 receptor (TIR) domain, and the external pathway, pointing away from the TIR domain. Remarkably, some ligands showed clear path preferences: the TLR8 agonist exited through the external unbinding pathway only, while the cationic antagonists exited through the internal pathway only. The neutral antagonists used both pathways. The mechanistic insights obtained can assist in the design of improved TLR modulators.

## Graphical abstract

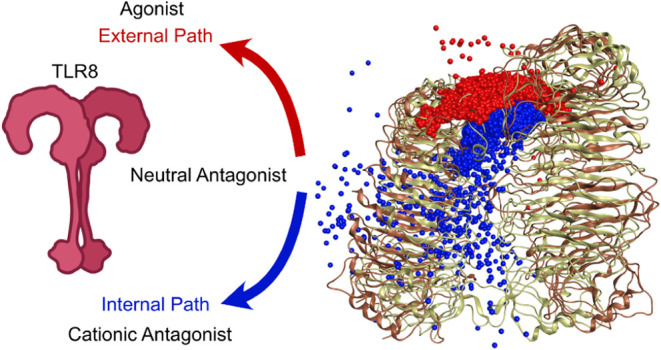

## Introduction

Toll-like receptors (TLRs) are a part of the innate immune response as they are responsible for the recognition of pathogen-associated molecular patterns (PAMPs) of viruses, fungi, parasites, and bacteria.^1,2^ Currently, there are 10 known TLRs in the human body, with TLR1, 2, 4, 5, 6, 10 located on the cell membrane surface, whereas TLR3, 7, 8, 9 are intracellular, located on endosomal membranes.^3–5^ Endosomal TLRs recognize double-stranded RNA (TLR3), single-stranded RNA (TLR7, 8), or single-stranded DNA (TLR9). TLR8, the focus of this work, is involved in the pathogenesis of diseases such as rheumatoid arthritis, systemic lupus erythematosus, and systemic sclerosis.^6–9^ Therefore, a mechanistic understanding of TLR8 ligand binding and inhibition may help in the development of therapeutics against autoimmune diseases.^10^

TLR8 is primarily expressed in monocytes, macrophages, and myeloid dendritic cells and is responsible for recognizing uridine-rich single-stranded RNA (ssRNA) from viruses and bacteria.^11^ TLR8 is a homodimer and has two conformations, active and inactive, both with experimental structures of their ectodomains available.^12,13^ In the active conformation, the homodimers are in closer proximity to each other compared to those in the inactive conformation, with the C-termini distance being 30 Å in the active conformation and 53 Å in the inactive conformation. The transition from the inactive to the active conformation of TLR8 is achieved through the binding of TLR8 to agonists, while TLR8 antagonists stabilize TLR8 in its inactive conformation.^12,13^

Previous studies employed molecular dynamics (MD) simulations to investigate the TLR conformation and interaction with modulators. Choudhury et al. used docking and MD simulations to investigate the binding modes of severe acute respiratory syndrome coronavirus 2 (SARS-CoV-2) mRNAs to intracellular TLRs 3, 7, 8, and 9.^14^ Shen et al. combined docking and MD simulations to characterize the interaction between chitin and TLR2.^15^ Ghosh et al. applied MD simulations to obtain insights into TLR1-TLR2 and TLR2-TLR6 dimer interaction patterns.^16^ Gosu et al. applied MD simulations to study the dynamic behavior of TLR3-dsRNA complexes.^17^ Hossen et al. applied MD simulations for the in silico design of peptides that could potentially act as TLR5 agonists.^18^ Ahmad et al. applied MD simulations in combination with deep learning to obtain additional insights into the mechanism of binding of Tomaralimab to TLR2.^19^

With respect to the research of TLR8, recent studies have employed MD simulations to investigate TLR8 and identify and optimize TLR8 agonists and antagonists. Sribar et al. performed a virtual screening campaign with structure-based 3D pharmacophores of TLR8 agonists in combination with molecular docking, which led to the discovery of novel pyrimidine-based TLR8 inhibitors.^20^ These pyrimidine-based compounds were then optimized in the work by Dolsak et al. and were analyzed through the application of molecular docking and MD simulations, followed by protein–ligand interaction analysis through the application of Dynophores.^21–23^ In the work of Sadeghkhani et al., pharmacophore models were developed and used for a virtual screening campaign with the hits of the screening undergoing molecular docking to obtain potential TLR8 agonists. MD simulations were performed to validate the molecular docking results, and free energy calculations were performed on the protein–ligand complexes of the best hits.^24^ An important application of MD simulations in the study of TLR8 was also performed by Bzówka et al., who applied MD simulations to obtain insights into the cleavage of the TLR8 Z-loop.^25^ However, there is a knowledge gap in the mechanism of unbinding of TLR8 agonists and antagonists.

## Results and Discussion

In this study, we aimed to cover this gap by investigating the unbinding pathways of one agonist and five antagonists of TLR8 using MD simulations and the τ-Random Acceleration Molecular Dynamics (τRAMD) method.^26,27^ τRAMD retrieves ligand unbinding events and relative residence times for a group of ligands interacting with the same protein.^28–30^ All the TLR8 agonist and antagonists investigated occupy the uridine binding site^12^ (Figure 1A) and have crystal structures for the complex with TLR8 available (PDB IDs: 3W3L, 5WYX, 6KYA, 6TY5, 7CRF, and 7YTX^12,13,31–34^ ). The antagonists could be categorized into two groups, depending on the absence or presence of a charged amine: the cationic antagonists, consisting of ligands **2** and **3**, and the neutral antagonists, ligands **4**, **5**, and **6** (Figure 1B).

**Figure 1:**
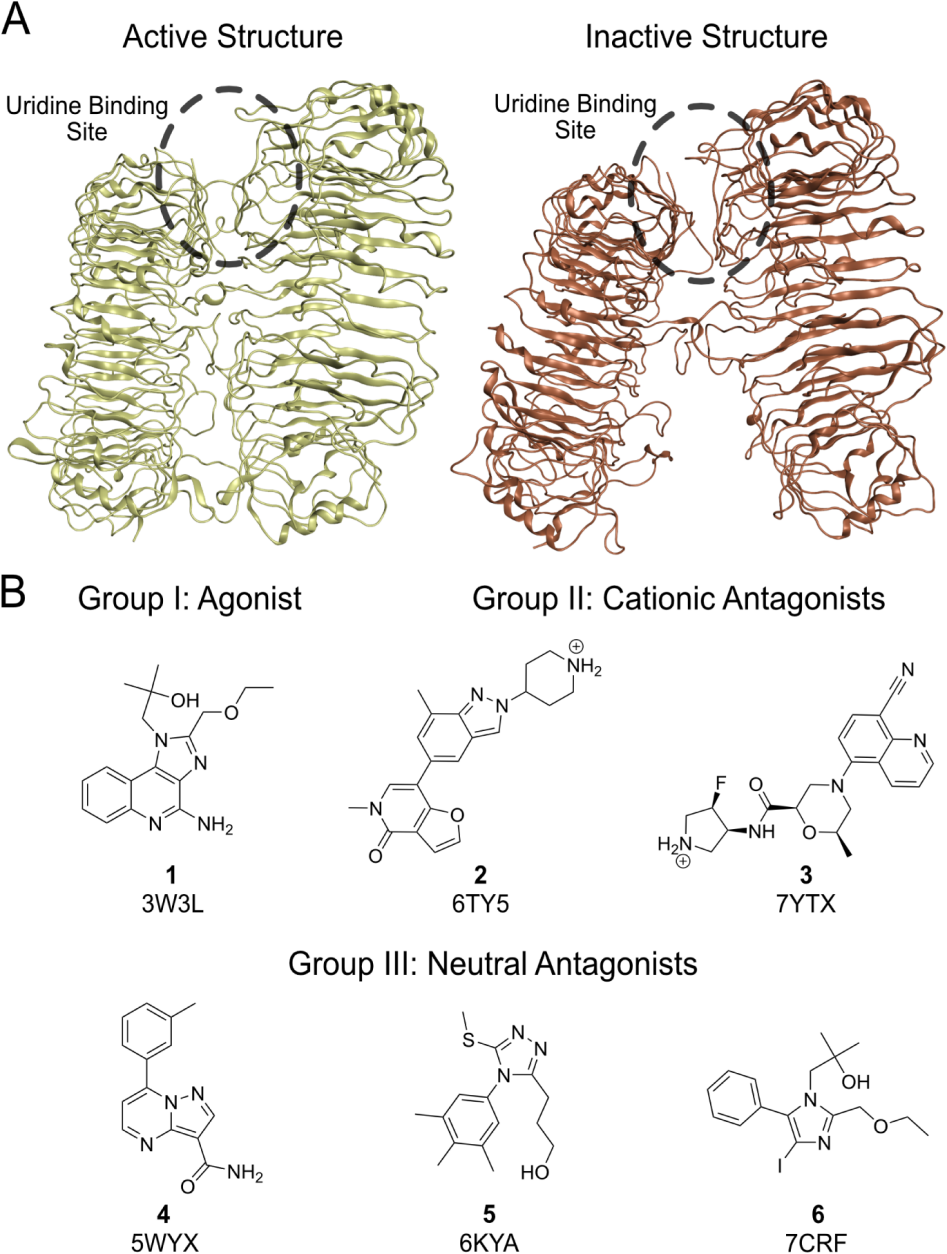
Structures of Toll-like receptor 8 in different states and of the ligands investigated. A) Structure of the Toll-like receptor 8 in the active and inactive states (PDB IDs: 3W3L and 6TY5 ^12,32^ ). B) Chemical structures of the ligands used in the simulations with their corresponding PDB IDs for the experimental structure of the receptor–ligand complex.^12,13,31–34^

### Two Unbinding Pathways Were Identified

After the equilibration of the protein–ligand complexes, τRAMD simulations were performed, resulting in 100 independent trajectories with unbinding events for every TLR8–ligand complex simulated. Next, a data analysis of the simulations was performed to identify the unbinding pathways and associated probabilities. Two possible unbinding pathways were identified by visual inspection of trajectories, and they were named as the internal path, which is directed toward the Toll-interleukin-1 receptor (TIR) domain and the cytoplasmic region, and the external path, which is directed away from the TIR domain, depending on the direction the ligand took to exit the uridine binding site (Figure 2A). The classification of unbinding paths for each trajectory was performed through the calculation of the minimum distance of the ligand to representative residues located next to the exit sites of the internal or external unbinding path, considering the final frames of the simulations (Figure S1). Remarkably, analysis of the pathway probabilities revealed that the TLR8 agonist **1** only undertook the external path for unbinding, while the cationic antagonists **2** and **3** only employed the internal path for unbinding (Figure 2B). The neutral antagonists **4–6** took either the external or internal path for unbinding (Figure 2B). A chi-square test indicated that the path probabilities for ligand **4** are not significantly different from random (50%:50%), while the path probabilities for ligands **5** and **6** are significantly different from random (*p*-values of 0.11, 3.2 × 10^–3^, and 2.7 × 10^–5^ for ligands **4**, **5**, and **6**, respectively). Ligands **5** and **6** show a slight preference for the external and internal path, respectively.

**Figure 2:**
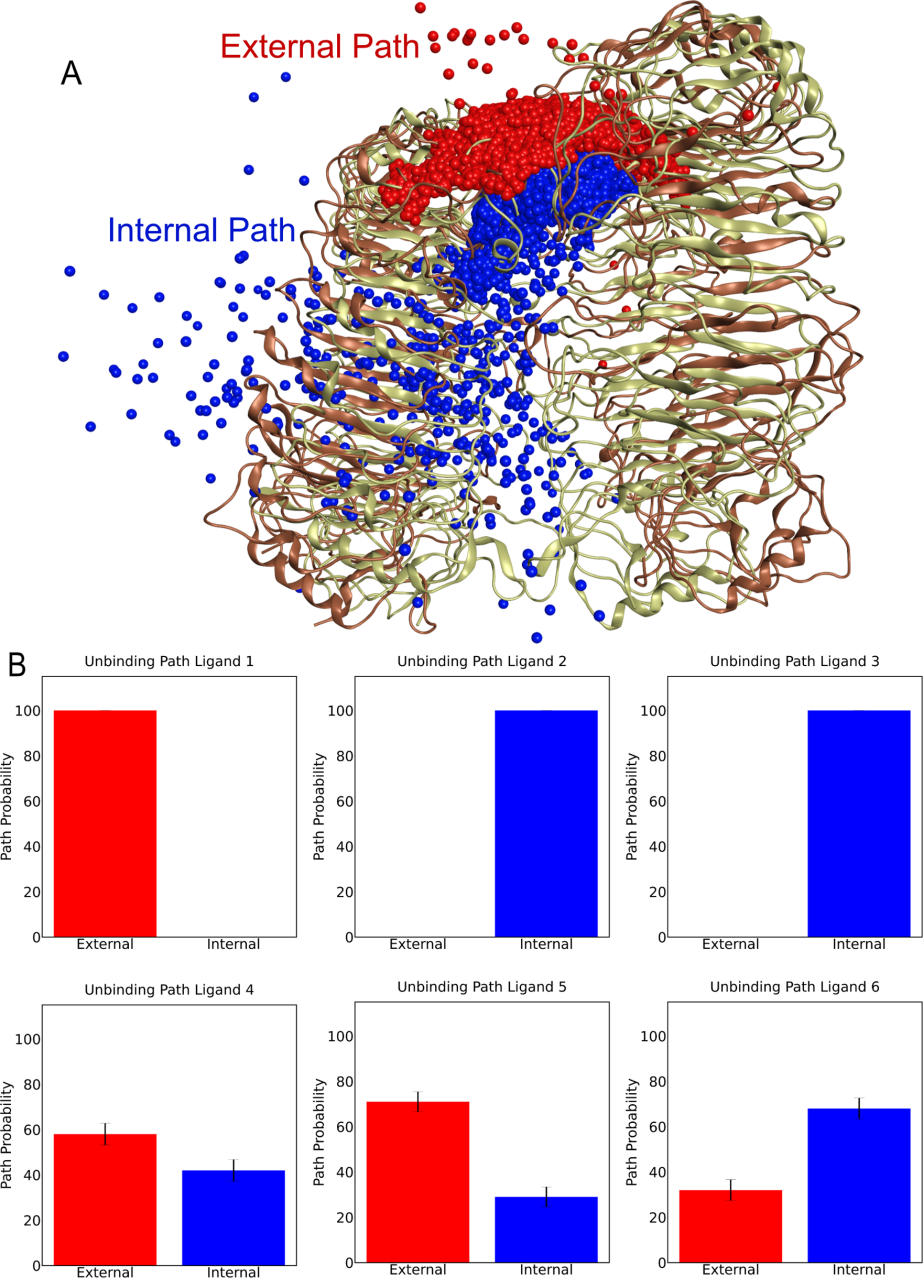
Toll-like receptor 8 ligand unbinding pathways and associated probabilities. A) Structure of the Toll-like receptor 8 in the active and inactive states (light brown: active structure, dark brown: inactive structure, PDB IDs: 3W3L and 6TY5 ^12,32^ ) with the external and internal unbinding paths represented as spheres, which were obtained through the calculation of the center of mass of the ligand during the simulation.^35,36^ In the internal path, the ligand moves toward the Toll-interleukin-1 receptor (TIR) domain, which is in the intracellular part of the receptor and not present in the crystal structures, while in the external path, the ligand moves away from the TIR domain. B) Probabilities with which the ligands undergo the external or internal unbinding paths in the τRAMD simulations. For each ligand, 100 unbinding events were obtained. The bars show standard errors obtained from the bootstrap analysis. A chi-square test indicated that the path probabilities for ligand **4** are not significantly different from random (50%:50%), while the path probabilities for ligands **5** and **6** are significantly different from random (*p*-values of 0.11, 3.2 × 10^–3^, and 2.7 × 10^–5^ for ligands **4**, **5**, and **6**, respectively).

The crystal structures and unbinding trajectories were visually inspected to investigate why the agonist and cationic antagonists employed a single type of path to leave the binding site. In the crystal structure of the active TLR8 (PDB ID: 3W3L), the agonist **1** forms hydrogen bonds with the side chain of residue Asp543* (with the asterisk denoting chain B) and with the backbone of Thr574*. Residues Tyr353 and Asp545* are located close to agonist **1**, sterically preventing it from exiting the binding site through the external path, while residues Arg429 and Tyr567* sterically prevent ligand **1** from exiting through the internal path (Figure 3A). The rotation of Tyr353 in the trajectories created space for ligand **1** motion, serving as a precursor for ligand **1** to exit the binding site through the external path (Figure 3B). Comparison between the initial and final frames of the τRAMD trajectories shows that there is a shift in the chi angles displayed by Tyr353 in the final frames (Figure S2), further supporting the importance of the rotation of Tyr353 for ligand unbinding. Additionally, a preliminary free energy profile for the motion of Tyr353 was calculated by using equilibrium MD simulations of the TLR8–ligand **1** complex. The preliminary free energy profile shows that there are two energy minima (Figure S3). The protein configuration with the rotation of Tyr353 required for ligand unbinding has a higher energy, being therefore less frequent in the simulations and was not sampled during the equilibrium MD simulations. Taken together, these results suggest that the unbinding of agonist **1** via the external path is facilitated by the rotation of Tyr353, explaining the preference of agonist **1** for this path.

**Figure 3:**
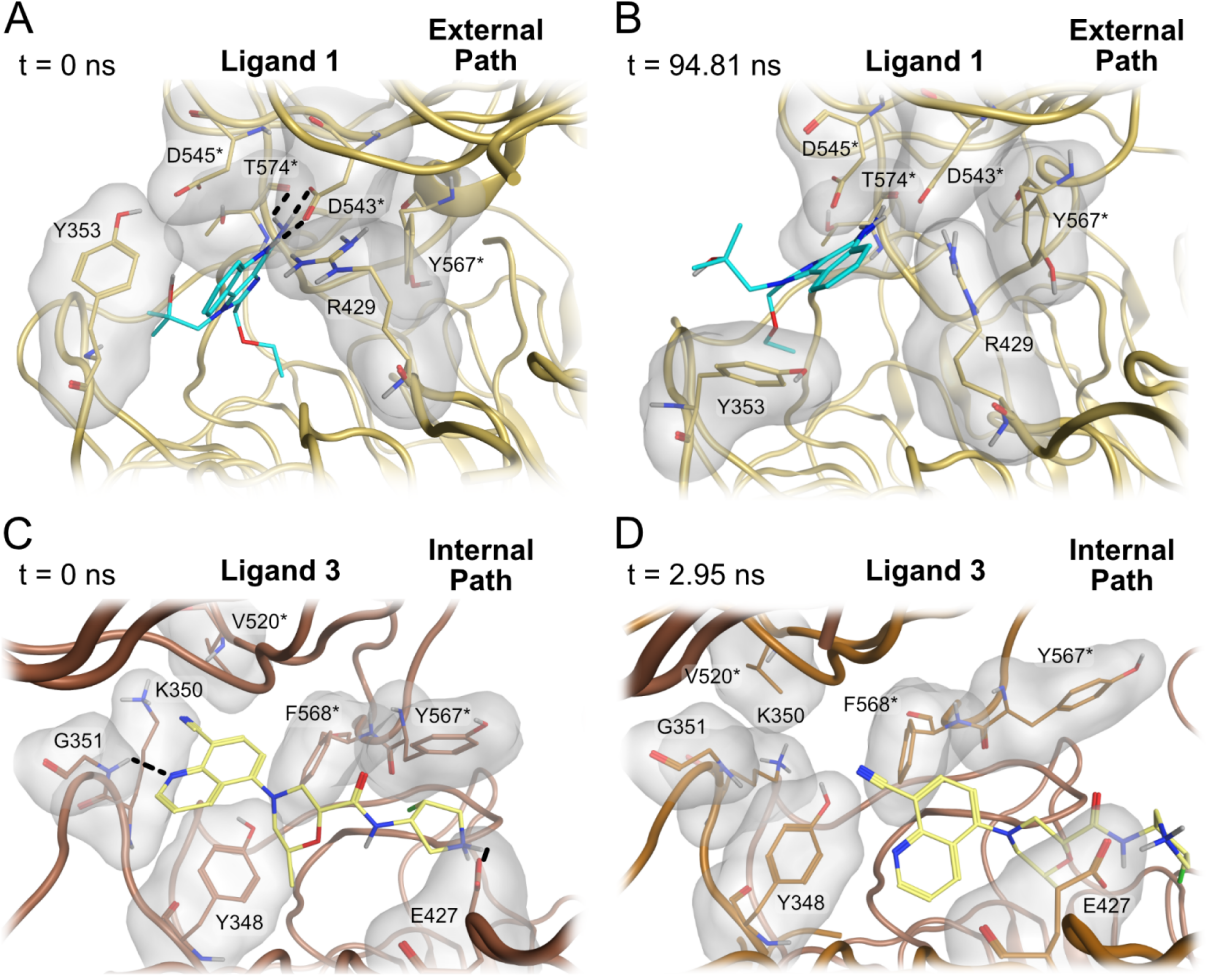
Main steps for the agonist and cationic antagonist unbinding process. A) Binding site of TLR8 agonist **1** (PDB ID: 3W3L^12^) at the start of the simulation. Ligand **1** forms hydrogen bonds with Asp543* and Thr574*. B) Binding site of TLR8 during the unbinding of **1**. The rotation of Tyr353 facilitates the unbinding of **1**. C) Binding site of TLR8 cationic antagonist **3** (PDB ID: 7YTX^34^) at the start of the simulation. **3** forms a hydrogen bond with the backbone of Gly351 and an ionic interaction with Glu427. D) Binding site of TLR8 during the unbinding of **3**. The motion of Tyr567* facilitates **3** unbinding. Light brown atoms and ribbon—active TLR8 protein structure, dark brown atoms and ribbon—inactive TLR8 protein structure, turquoise atoms—TLR8 agonist **1**, and yellow atoms—cationic antagonist **3**. Hydrogen bonds are indicated as black traced lines. Gray surfaces indicate the molecular surface of the displayed residues near the ligand.

The cationic antagonists **2** and **3** showed behavior different from the agonist **1** in the simulations. In the crystal structure 7YTX, antagonist **3** displays a hydrogen bond with the backbone of residue Gly351, while simultaneously forming an ionic interaction with the side chain of residue Glu427. The antagonist is sterically hindered from exiting through the external path by the residues Lys350, Gly351, and Val520*, while the residues Glu427 and Tyr567* sterically hinder it from exiting the binding site through the internal path (Figure 3C). In some simulations, motion of the side chain of residue Tyr567* allowed the antagonist to exit the binding site through the internal pathway (Figure 3D). For cationic antagonists, unbinding via the internal path is facilitated by the motion of Tyr567*, explaining the preference of cationic antagonists for this path. When TLR8 is bound to neutral antagonists, Tyr567* forms a hydrogen bond with Glu427, which hinders the motion of Tyr567* and, therefore, makes ligand unbinding via the internal path more difficult (see below). This hydrogen bond is not formed in the structures of cationic antagonists due to the formation of an ionic interaction of Glu427 with the antagonists.

Finally, the simulations with the neutral antagonists **4**, **5**, and **6** were also visually inspected to identify the main motions during unbinding through the internal and external paths. The neutral antagonists **4**, **5**, and **6** form a hydrogen bond with the backbone of Gly351, identical to the cationic antagonists, but they lack the ionic interaction with Glu427. The lack of this interaction allows the formation of a hydrogen bond between the side chain of Glu427 and the side chain of Tyr567*, which sterically hinders **4** from exiting the binding site via the internal path, while residues Lys350, Gly351, and Val520* sterically hinder **4** from exiting the binding site via the external path (Figure 4A). During the simulations, Lys350 and Gly351 move away from Val520*, allowing **4** to exit through the external path due to the increased space (Figure 4B). The external path of neutral antagonists presents small differences in comparison to the path of agonist **1** due to the structural differences between active and inactive TLR8. While Tyr353 plays an important role in guiding the unbinding of **1** via the external path, it is less involved in the external path of neutral antagonists. Alternatively, due to the hydrogen bond between Glu427 and Tyr567* (Figure 4C), rotation of Phe568* was required for ligand **4** to move closer to Phe320, Phe470, and Phe568* (Figure 4D). Subsequently, **4** moved closer to Phe461 and Thr471 (Figure 4E), resulting in unbinding through the internal path. The side chain of Phe461 sterically prevents **4** from exiting the binding site; thus, an additional rotation of the Phe461 side chain is required for complete ligand unbinding (Figure 4F).

**Figure 4:**
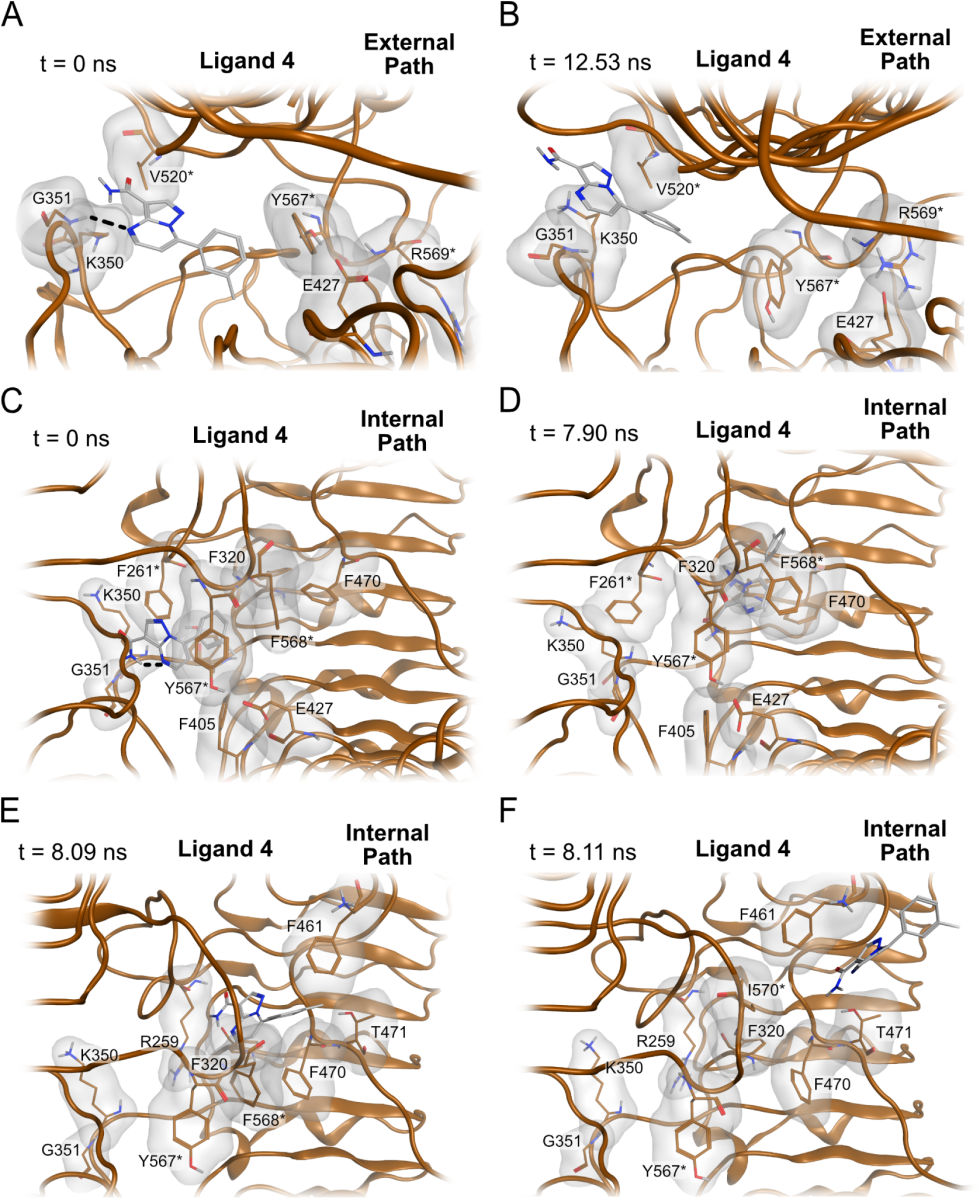
Main steps for the neutral antagonist unbinding process. A and B represent unbinding through the external path, C–F represent unbinding through the internal path. A) Binding site of neutral TLR8 antagonist **4** (PDB ID: 5WYX^13^) at the start of the simulation. B) Binding site of **4** during the exit via the external path. Lys350 and Gly351 move away from Val520*, thus creating enough space for **4** to exit the binding site. C) Binding site of **4** at the start of the simulation, with Glu427 and Tyr567* forming a hydrogen bond. D) **4** loses its hydrogen bond with the backbone of Gly351 and moves closer to residues Phe320, Phe470, and Phe568*. E) **4** is located next to Phe461 and Thr471, with the side chain of Phe461 preventing **4** from exiting the binding site. F) The rotation of the Phe461 side chain allowed **4** to exit the binding site via the internal pathway. Dark brown atoms and ribbon—inactive TLR8 protein structure and gray atoms—neutral antagonist. Hydrogen bonds are indicated as black traced lines. Gray surfaces indicate the molecular surface of the displayed residues near the ligand.

The results show that the neutral and cationic antagonists have a different behavior, with the cationic antagonists **2** and **3** unbinding through the internal path only, and the neutral antagonists **4**, **5**, and **6** unbinding through both paths, because **2** and **3** form an ionic interaction with Glu427, which prevents the side chain of Glu427 from forming the hydrogen bond with the side chain of Tyr567* observed in crystal structures with neutral antagonists **4**, **5**, and **6**. The lack of this hydrogen bond facilitates the exit of cationic antagonists through the internal path, while the neutral antagonists can undergo both possible unbinding paths.

Mutational studies were performed to validate the hypothesis that the hydrogen bond between the side chain of Glu427 and Tyr567* blocks the internal path for neutral antagonists **4**, **5**, and **6**, leading, therefore, to a lack of clear path preference for these antagonists. A Y567F* mutation was introduced in TLR8, and τRAMD simulations were performed for the mutant in complex with antagonist **5**. The expectation was that this mutation would prevent the formation of the hydrogen bond between Tyr567* and Glu427, thereby facilitating unbinding through the internal path. Unexpectedly, ligand **5** still exhibited unbinding through both the internal and external paths. This is explained by the formation of an ionic interaction in the mutant Y567F* between Glu427 and Arg569* in four out of five replicas during the equilibration simulations, which led to hindrance of the internal unbinding pathway (Figures S4 and S5).

In order to disrupt all hydrogen bonds and ionic interactions that could be formed by Glu427 and to properly test the role of the hydrogen bond between the side chain of Glu427 and Tyr567* in the preference for the internal path, we introduced the mutation E427A in TLR8 and performed τRAMD simulations for the mutant in complex with the neutral antagonist **5**. The E427A mutation prevented the formation of the hydrogen bond between Glu427 and Tyr567* and the ionic interaction between Glu427 and Arg569*. For this mutant, we obtained the expected results, and the neutral antagonist **5** exited via the internal unbinding pathway in all τRAMD simulations (Figures 5 and S6). These results confirm our initial observation that the hydrogen bond between Glu427 and Tyr567* modulates the ligand pathway preference in antagonists.

**Figure 5:**
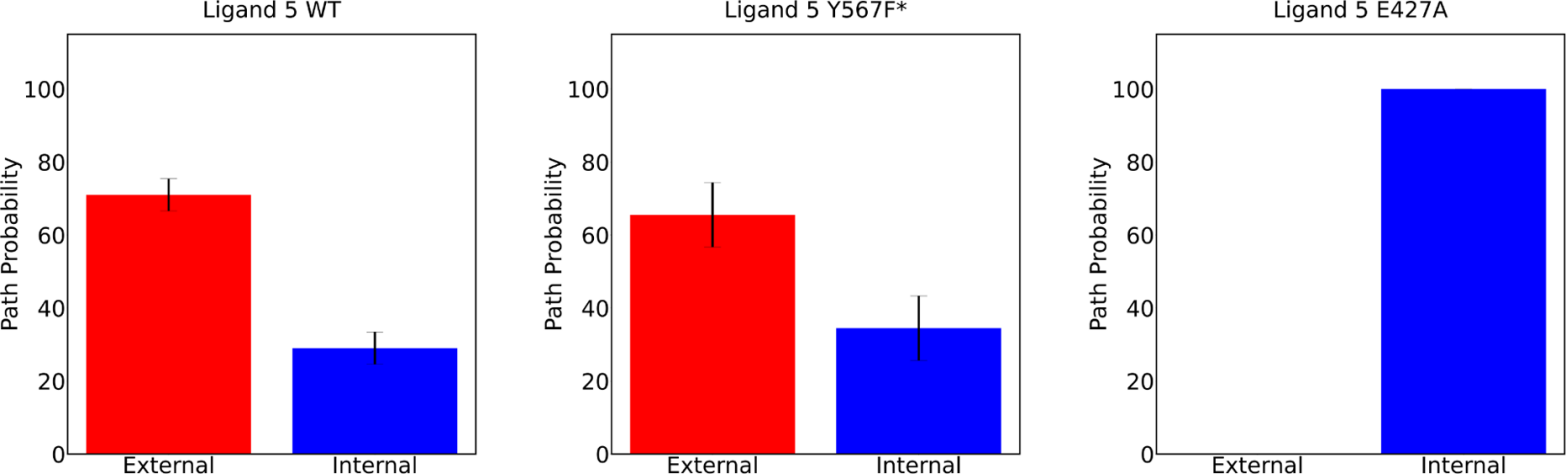
Probabilities with which ligand **5** dissociates from TLR8 (wild-type (WT), mutant Y567F*, or mutant E427A) via the external or internal paths in the τRAMD simulations. For each TLR8 form, 100 (WT), 29 (Y567F*), and 18 (E427A) unbinding events were obtained. The bars show standard errors obtained from bootstrap analysis.

### The Agonist Displays Longer Residence Times Compared to Antagonists

Table 1 compares the residence times obtained from τRAMD simulations for the agonist **1** and antagonists **2–6** (further information in Figures S7–S18). The differences among the residence times are statistically significant (one-way ANOVA, *p*-value: 4.2e-38). The agonist **1** displayed on average a longer residence time of 40.0 ns, while the antagonists **2–6** displayed an average residence time of 4.5–14.3 ns. A possible reason for the agonist’s longer residence time can be its unbinding via the external path only. Moreover, neutral antagonists were able to unbind via both pathways. The presence of two possible unbinding paths, instead of one, likely contributed to the reduced residence times for neutral antagonists in comparison to the agonist.

**Table 1:** Residence Times (Average and Standard Deviation) of the TLR8–Ligand Complexes Computed from τRAMD Simulations

Ligand	Average Residence Time [ns]	Standard Deviation [ns]
1	40.0	11.8
2	7.7	2.2
3	9.7	9.1
4	8.8	3.4
5	14.3	5.3
6	4.5	2.8

## Conclusion

This study used MD simulations in combination with τRAMD to elucidate for the first time the mechanism of dissociation of agonists and antagonists of TLR8. Two unbinding pathways were identified: the external and internal paths. Surprisingly, some ligands showed clear preferences for one specific unbinding path in our simulations. The agonist dissociated only through the external path, while the cationic antagonists dissociated only through the internal path. Neutral antagonists were able to dissociate through both the internal and external paths. These differences in path preference are explained by the protein–ligand interactions in the crystal structures, especially differences between cationic and neutral antagonists. Cationic antagonists present an ionic interaction with Glu427, which prevents the side chain of Tyr567* from forming a hydrogen bond with Glu427, thus leaving more space for the antagonist to exit through the internal path. The different pathways can be of physiological relevance. The internal unbinding pathway points toward the cell membrane. This path may increase the chances of protein–ligand rebinding due to the presence of the membrane as a barrier for free diffusion of the ligand. Increased chances of rebinding may, in turn, lead to longer physiological effects. Additionally, in contrast to the antagonists, the agonist presented longer residence times. This may be due to its preference for unbinding via the external path. Future studies can expand on the methods and analyses employed here to investigate the ligand unbinding pathways of other Toll-like receptors. An important aspect would be to study the behavior of diverse TLR agonists to determine if they display the same path preference and longer residence times as observed here for the agonist of TLR8. The mechanistic insights provided here could be exploited to design antagonists with an unbinding pathway similar to the agonist, displaying longer residence times, or to design antagonists with a preference for the internal path, such as the cationic antagonists, which can increase the chances of rebinding.

## Data and Software Availability

The input files and starting structures for MD simulations, data, and results of the study can be found at: https://github.com/talagayev/TLR8_TauRAMD

## Methods

### Protein Structure Preparation

We selected TLR8–ligand complexes with experimental structures and experimental dissociation constants available. Six complexes were chosen according to these criteria (PDB IDs: 3W3L, 5WYX, 6KYA, 6TY5, 7CRF, and 7YTX^12,13,31–34^ ). The crystal structures were retrieved from the RCSB PDB. The buffer additives were removed, while the crystallographic waters in a proximity of 5 Å to the cocrystallized ligand and the ligand were retained. The structure preparation was performed in MOE 2022 (Chemical Computing Group, Montreal, Canada). The missing loops (PDB 3W3L: chain A and chain B: residues 101–112; PDB 5WYX: chain A: residues 101–111, chain B: residues 100–111, 568–572, and 755–761; PDB 6KYA: chain A: residues 101–111 and 755–761, chain B: residues 101–111, 728–736, and 754–763; PDB 6TY5: chain A: residues 100–112, 184–186, and 625–628, chain B: residues 100–112 and 183–186; PDB 7CRF: chains A and B: residues 100–111 and 753–762; PDB 7YTX: chain A: residues 101–111, chain B: residues 101–111 and 756–761) were modeled with the Loop Modeler utility present in MOE, except for the Z-loop, which is located between the leucine-rich repeat (LRR) 14 and LRR15.^37^ The Z-loop is cleaved in vivo before protein dimerization, and the residues removed from the structures in vitro can vary depending on the experimental group (PDB 3W3L: residues 434–458 for chains A and B; PDB 5WYX: residues 434–460 for chain A, residues 436–460 for chain B; PDB 6KYA: residues 435–460 for chains A and B; PDB 6TY5: residues 435–461 for chains A and B; PDB 7CRF: residues 437–459 for chains A and B; PDB 7YTX: residues 438–460 for chains A and B). Residue 601 was missing in both 6KYA chains, and residue 734 was missing in chain A. Thus, residues 599–601 were modeled in both chains, and residues 733–735 were modeled in chain A due to the restriction of a minimum of three residues required for the Loop Modeler in MOE. Modeling of missing side chains and capping was performed using the Structure Preparation utility. The protein was protonated using the Protonate 3D function^38^ at pH 7 and a temperature of 300 K. The mutations in the mutated structures (mutants E427A and Y567F*) were introduced using the protein builder function implemented in MOE. The residue was mutated, which was followed by an energy minimization of the side chain of the mutated residue and the tethered backbones.

### Ligand Parameterization

The τRAMD simulations were carried out using GROMACS-RAMD version 2.0,^27,39–41^ the AMBER14SB force field,^42^ and the general Amber force field (GAFF)^43^ for protein and ligands, respectively. The ligand partial charges were calculated with quantum mechanical (QM) calculations using Gaussian 09,^44^ Hartree–Fock,^45^ and the 6–31G* basis set for all ligands except the neutral antagonist 6 of the crystal structure 7CRF,^33^ which contained an iodine. Due to the σ-hole of the iodine in ligand **6**, and following previous work,^46–51^ we decided to use a higher basis set for QM calculations for ligand **6**. For this ligand, the LANL2DZdp^49,52^ basis set was retrieved from the basis exchange server and applied. AmberTools 24 ^53^ was used to generate the topology and coordinate files of the ligand.

### Molecular Dynamics Simulations

Each protein–ligand complex was placed in a cubic TIP3P water^54^ box with a padding distance of 1 nm to the surface of the protein. Sodium and chloride ions were added to neutralize the system and obtain an ionic concentration of 150 mM. Simulations for heating and pressure adjustment were performed with AmberTools 24,^53^ with each structure undergoing five separate simulations, resulting in five replicas. The complex was energy-minimized in four stages using the steepest descent method, with a maximum of 1500 steps and a gradual reduction of the positional restraints applied on the protein’s heavy atoms (500, 100, 5, 0 kcal/mol/Å^2^). In the next step, the energy-minimized system underwent an NVT ensemble heating procedure, with the temperature being increased from 10 to 300 K. Positional restraints of 50 kcal/mol/Å^2^ were applied to the heavy atoms during this step. The simulations were performed for 25 000 steps with a time step of 0.002 ps using the Nose–Hoover thermostat^55^ and Langevin dynamics with a friction coefficient of 1.0 ps^−1^. This was followed by an equilibration protocol consisting of four steps performed in an NPT ensemble with a constant temperature of 300 K using the Nose–Hoover thermostat,^55^ a constant pressure of 1 bar using the Berendsen barostat,^56^ and a gradual reduction of the positional restraints over the heavy atoms of the protein (50, 10, 2, 0 kcal/mol/Å^2^). Each step of the equilibration consisted of 10 000 steps, with a time step of 0.002 ps. van der Waals and electrostatic forces were computed by using a cutoff of 1.0 nm. Electrostatics beyond the cutoff were treated using the particle mesh Ewald (PME) method.^57,58^

The next simulations for replica equilibration and ligand unbinding were carried out with GROMACS-RAMD version 2.0.^27,39–41^ Each of the five replicas underwent an additional equilibration with an NVT ensemble with the Berendsen thermostat^56^ and a constant temperature of 300 K. The equilibration consisted of 10 000 000 steps with a time step of 0.002 ps, resulting in 20 ns simulation time. For the mutated structures of TLR8 (mutants E427A and Y567F*), the equilibration time was longer and consisted of 50 000 000 steps with a time step of 0.002 ps, resulting in a 100 ns simulation time. For the preliminary free energy profile, the equilibration time of TLR8 in complex with ligand **1** was extended and consisted of 250 000 000 steps with a time step of 0.002 ps, resulting in 500 ns simulation time. Electrostatic forces were calculated by using the PME method. This resulted in five equilibrated replicas, each serving as a starting point for 20 individual τRAMD simulations for ligand unbinding, resulting in 100 unbinding events for each complex. For the mutated structures of TLR8 in complex with ligand **5**, five replicas were obtained for mutant Y567F*. Three of the replicas were used for τRAMD simulations, with two of the replicas resulting in 10 unbinding events each and nine unbinding events in the remaining replica, resulting in a total of 29 unbinding events. Five replicas were obtained for mutant E427A. Two of those replicas were used for τRAMD simulations. Eight unbinding events were obtained from each of the replicas, resulting in 16 unbinding events. The τRAMD simulations were performed with an NPT ensemble, the Nose–Hoover thermostat with a constant temperature of 313 K, and the Parrinello–Rahman barostat with a constant pressure of 1 bar. The maximum duration of the simulations was 50 000 000 steps, with a time step of 0.002 ps, resulting in a maximum simulation time of 100 ns. The van der Waals forces were computed using a cutoff of 1.2 nm. Electrostatic forces were calculated using the PME method with a real-space cutoff of 1.2 nm, a PME order of four, and a Fourier grid spacing of 1.2 Å. The linear center of mass motion of the protein and ligand was removed every 100 steps of the simulation. The covalent bonds to hydrogens in the solute were constrained by using the LINCS algorithm. For the τRAMD parameters, the magnitude of the force applied to the ligands during the simulations was 9 kcal/mol/Å, the threshold distance was set to 0.0025 nm, and the evaluation frequency was set to 50 fs. The reference atom of the protein was the backbone amine of G351. An unbinding event was identified when the ligand showed a distance of >6 nm from the reference atom.

### Simulation Analysis

The analysis of the trajectories was performed with MDAnalysis.^35,36^ For the categorization of the trajectories into the internal and external pathways, the final 10 frames of the simulation were used for the calculation of the minimum distance of the ligand to representative residues located next to the exit sites of the internal or external path (ligand **1**: external path: Asp545*, internal path: Asn491; ligands **2** and **3**: external path: Phe526*, internal path: Asn491; ligands **4**, **5**, and **6**: external path: Phe526*, internal path: Ala571*). The trajectory was attributed to the path associated with the representative residue with the lowest distance from the ligand (Figure S1). The protein–ligand interactions along the unbinding pathways were different for the agonist **1**, cationic antagonists **2** and **3**, and the neutral antagonists **4**, **5**, and **6**; thus, different pairs of residues were used for the distance calculation. Some trajectories presented minimum distances similar to those of the representative residues of both paths (points close to the dotted lines in Figure S1) and were further inspected visually to avoid classification errors (points highlighted with circles in Figure S1). Some trajectories presented residue–ligand distances longer than expected due to problems with positioning the whole protein inside the simulation box and were also further inspected visually to avoid classification errors (points highlighted with circles in Figure S1). The graphs for the visualization of the results were created with Matplotlib.^59^ In the simulations with mutated TLR8, MDAnalysis^35,36^ was used to calculate the minimum distance between the atoms of the residues Glu427 and Phe567* and the atoms of the residues Glu427 and Arg569*. For the generation of the preliminary free energy profile, PyEMMA^60^ was used. The pairwise distances between non-hydrogen atoms of residues Tyr353 and Asp545* were used as features. Time-lagged independent component analysis (TICA) was performed with the TICA lag time set to 10 ps. Matplotlib^59^ was used to generate the figures.

## Supplementary Material



## Notes

Scatter plots of minimum distances between the ligand and specific residues, which were used to assist unbinding pathway classification (Figure S1); Chi angle calculation of Tyr353 for the first and last five frames of τRAMD trajectories of ligand **1** (Figure S2); preliminary free energy profile of ligand **1** in equilibrium MD simulations (Figure S3); minimum distance between Glu427 and Phe567* in equilibration simulations for mutant Y567F* (Figure S4); minimum distance between Glu427 and Arg569* in equilibration simulations of mutant Y567F* (Figure S5); scatter plots of minimum distances between ligand **5** and specific residues for the mutants Y567F* and E427A (Figure S6); residence times of ligand **1** (Figure S7); distribution of residence times of ligand **1** (Figure S8); residence times of ligand **2** (Figure S9); distribution of residence times of ligand **2** (Figure S10); residence times of ligand **3** (Figure S11); distribution of residence times of ligand **3** (Figure S12); residence times of ligand **4** (Figure S13); distribution of residence times of ligand **4** (Figure S14); residence times of ligand **5** (Figure S15); distribution of residence times of ligand **5** (Figure S16); residence times of ligand **6** (Figure S17); distribution of residence times of ligand **6** (Figure S18) (PDF)
