# Supplementary material for: Agonists and Antagonists Show Different Unbinding Paths from the TLR8 Receptor
Source: J Chem Inf Model. 2025 Jul 9;65(14):7678–88. doi: 10.1021/acs.jcim.5c00496 (PMC12308803; doi:10.1021/acs.jcim.5c00496)
Supplement: Supplementary file 1 [file ci5c00496_si_001.pdf]

Supplementary Information

Agonists and antagonists show different unbinding paths from the TLR8 receptor

Valerij Talagayev[1], Gerhard Wolber[1], Ariane Nunes-Alves[2*]*

[1]Department of Pharmaceutical and Medicinal Chemistry, Institute of Pharmacy, Freie Universität Berlin, Königin-Luise-Str. 2+4, 14195 Berlin, Germany

[2]Institute of Chemistry, Technische Universität Berlin, Straße des 17. Juni 135, 10623 Berlin, Germany

[*]Corresponding author: gerhard.wolber@fu-berlin.de or ferreira.nunes.alves@tu-berlin.de

Figure S1 - Scatter plots of minimum distances between the ligand and specific residues which were used to assist unbinding pathway classification.

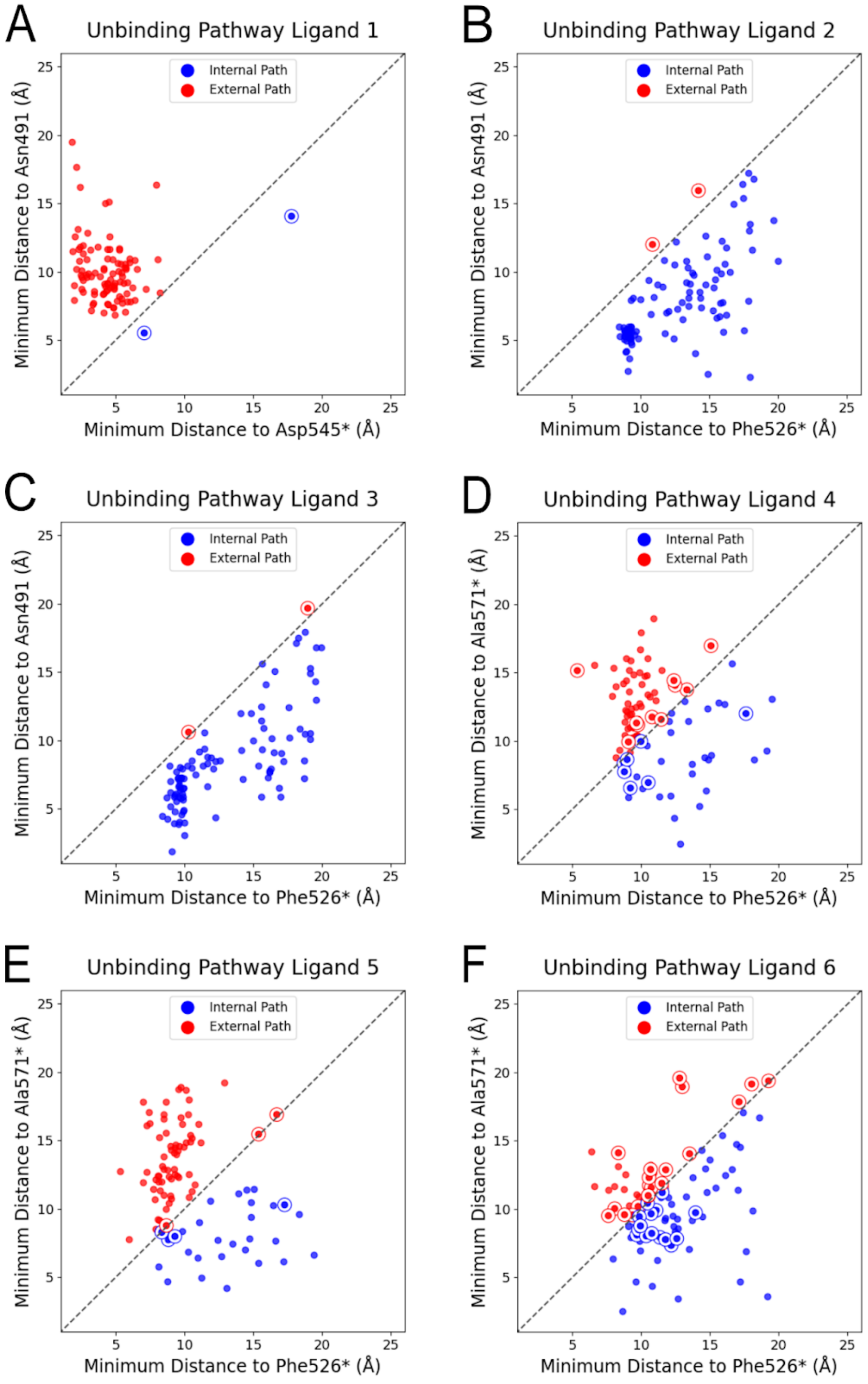

Figure S1. Scatter plots of minimum distances between the ligand and specific residues which were used to assist unbinding pathway classification. The classification of the trajectories into the unbinding pathways was performed through the calculation of the minimum distance between the ligands and a representative residue next to the internal or external path in the final 10 frames of the simulations. The trajectory was attributed to the path associated with the closest residue. A) For the agonist **1** Asn491 was used to represent the internal path and Asp545* was used to represent the external. B, C) For the cationic antagonists **2** and **3** Asn491 was used to represent the internal path and Phe526* was used to represent the external path. D-F) For the neutral antagonists **4**, **5** and **6** Ala571* was used to represent the internal path and Phe526* was used to represent the external path. The points that had their path classification modified after visual inspection (due to proximity to both representative residues, or due to problems to position the whole protein in the simulation box) are highlighted with circles.

Figure S2 - Chi angle calculation of Tyr353 for the first and last five frames of tauRAMD trajectories of ligand 1.

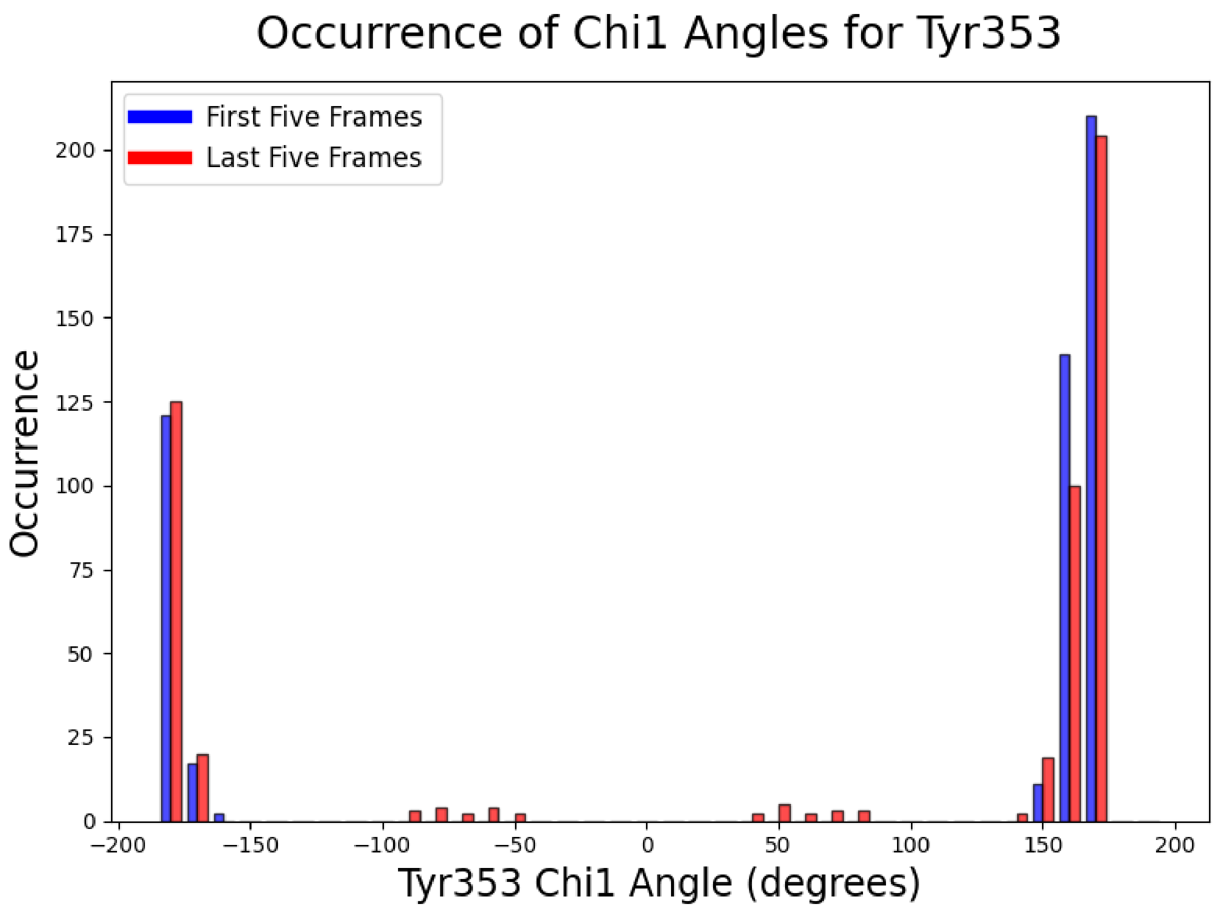

Figure S2. Chi angle calculation of Tyr353 for the first and last five frames of tauRAMD trajectories of ligand **1**. The population shift of the chi angles indicates the occurrence of the rotation of the side chain of Tyr353 during the trajectories and thus the importance of this rotation as a precursor for the unbinding through the external pathway for ligand **1**.

Figure S3 – Preliminary free energy profile of ligand 1 in equilibrium MD simulations.

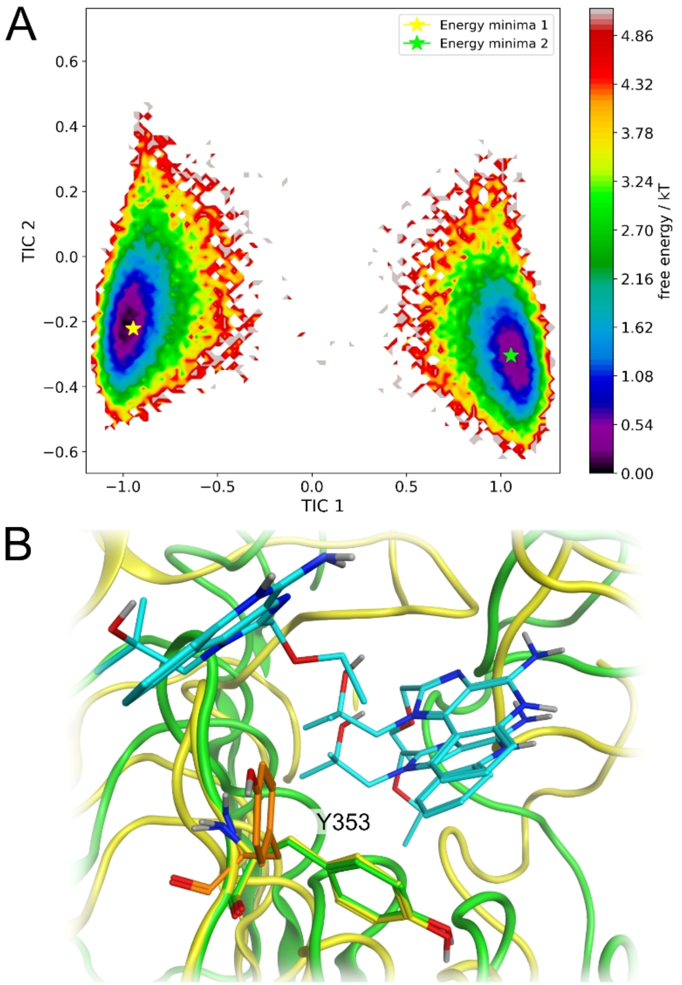

Figure S3. Preliminary free energy profile of agonist 1 bound to TLR8. A) Preliminary free energy profile from equilibrium MD simulations show two stable states. B) Representative snapshots of TLR8 in the different conformations highlighted in panel A with stars and the unbinding event, which was not sampled in the equilibrium MD simulations. Legend: green protein structure and residue atoms: energy minima 1, yellow protein structure and residue atoms: energy minima 2, orange protein structure and residue atoms: Tyr353 rotation frame displaying the change in the angle during the unbinding event. Cyan atoms: ligand **1**.

Figure S4 – Minimum distance between Glu427 and Phe567* in equilibration simulations for mutant Y567F*.

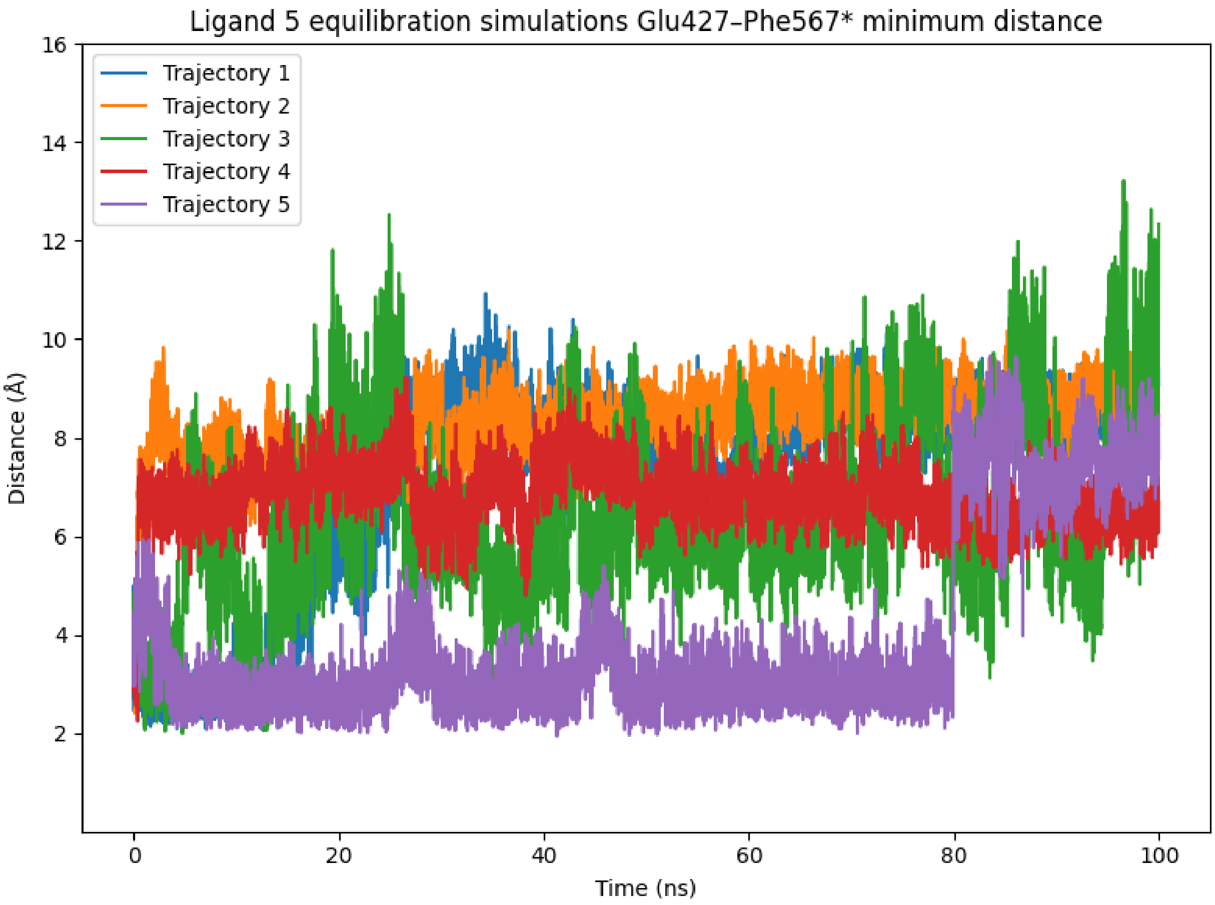

Figure S4. Minimum distance between Glu427 and Phe567* during equilibration simulations of the mutant Y567F* in complex with ligand 5. During the equilibration simulations the distance between the residues increases, indicating that the hydrogen bond present in the wild type protein is absent.

Figure S5 – Minimum distance between Glu427 and Arg569* in equilibration simulations of mutant Y567F*.

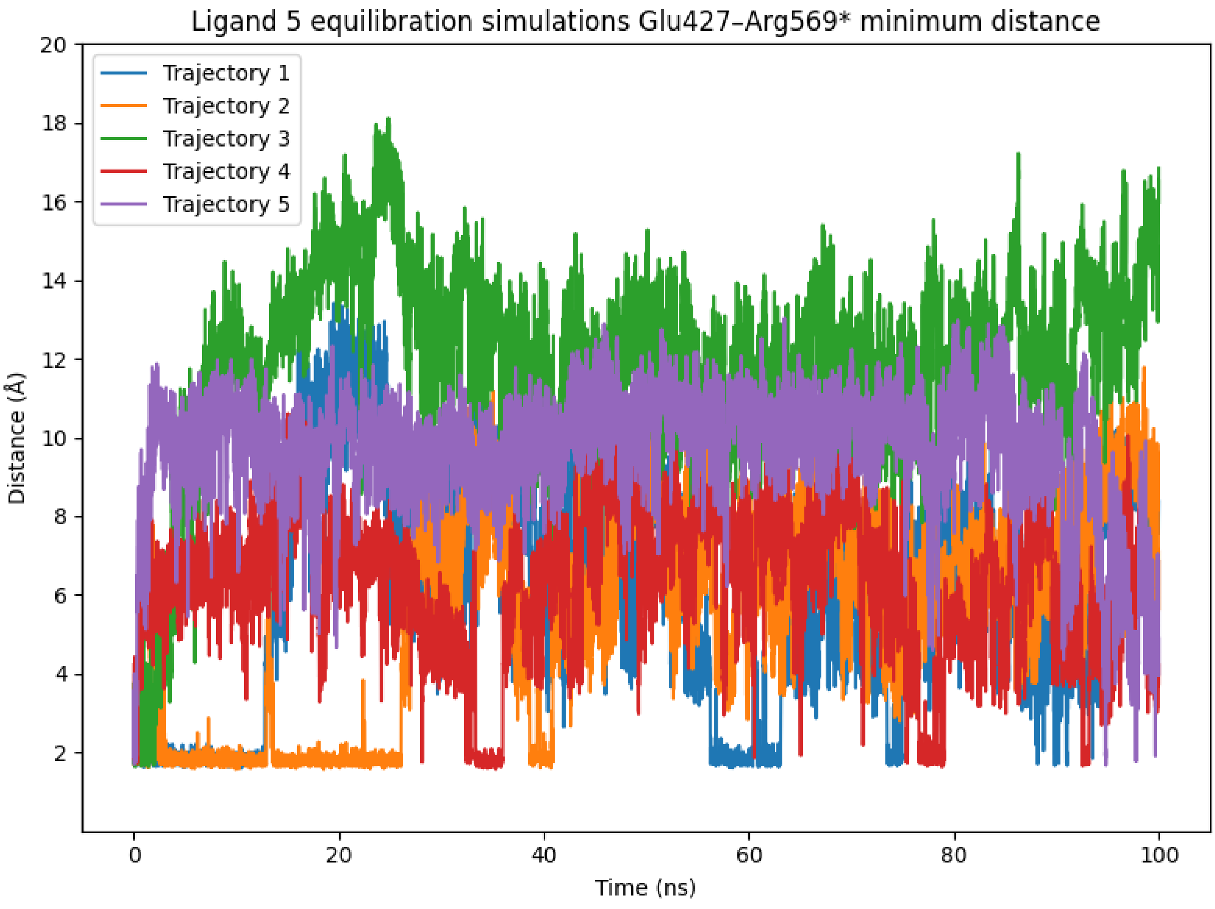

Figure S5. Minimum distance between residues Glu427 and Arg569* during equilibration simulations of mutant Y567F*. During the equilibration simulations the distance between the residues is around 2-8 Å, indicating the presence of an ionic interaction. Replica 3 showed an increase of the distance between Glu427 and Arg569*.

Figure S6 – Scatter plots of minimum distances between ligand 5 and specific residues for the mutants Y567F* and E427A.

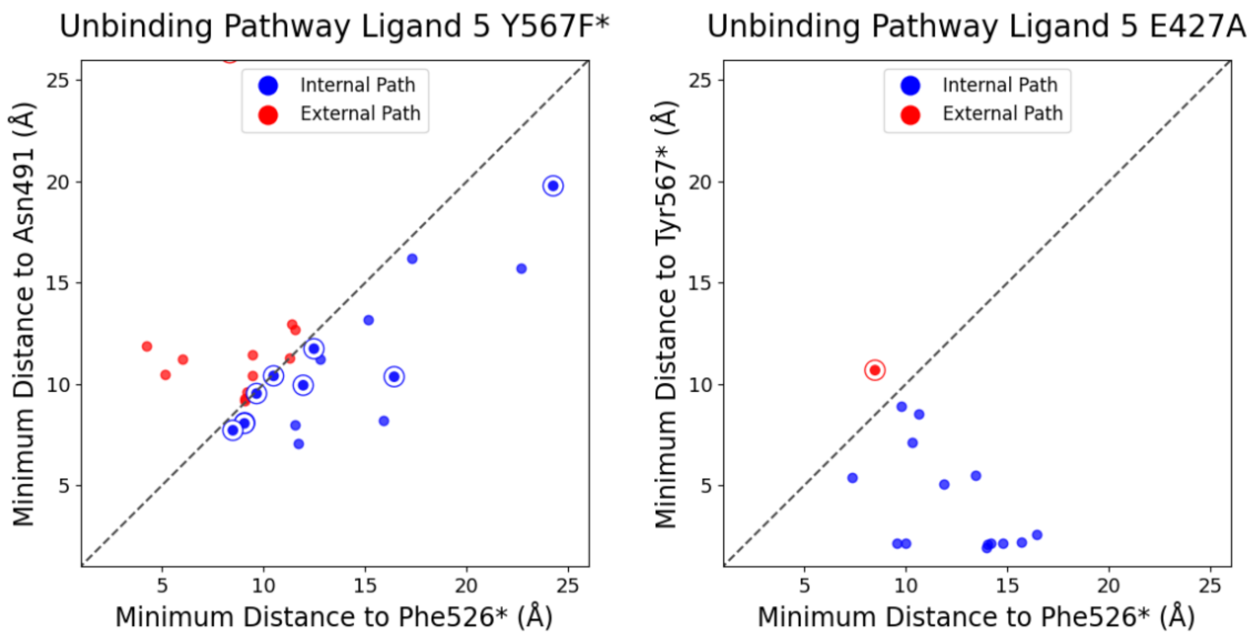

Figure S6. Scatter plots of minimum distances between the ligand and specific residues which were used to assist unbinding pathway classification of the Y567F* and E427A mutations. The classification of the trajectories into the unbinding pathways was performed through the calculation of the minimum distance between the ligands and a representative residue next to the internal or external path with the external residue Phe526* being used for the distance calculation for both mutations, while the internal residues Asn491 and Tyr567* were used for mutants Y567F* and E427A, respectively.

Figure S7 - Residence times of ligand 1.

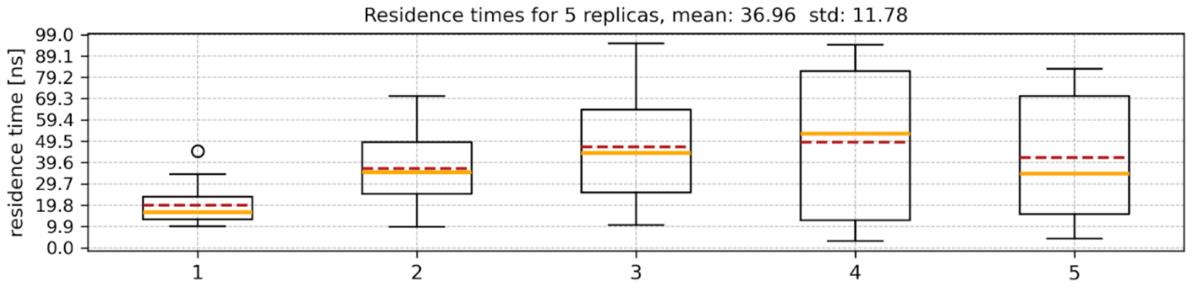

Figure S7. Residence times of ligand **1**. Box plots show the distribution of residence times obtained from tauRAMD trajectories of ligand **1** in five replicas (20 trajectories per replica). The yellow line displays the mean time, while the red dotted line shows the median of the residence times.

Figure S8 - Distribution of residence times of ligand 1.

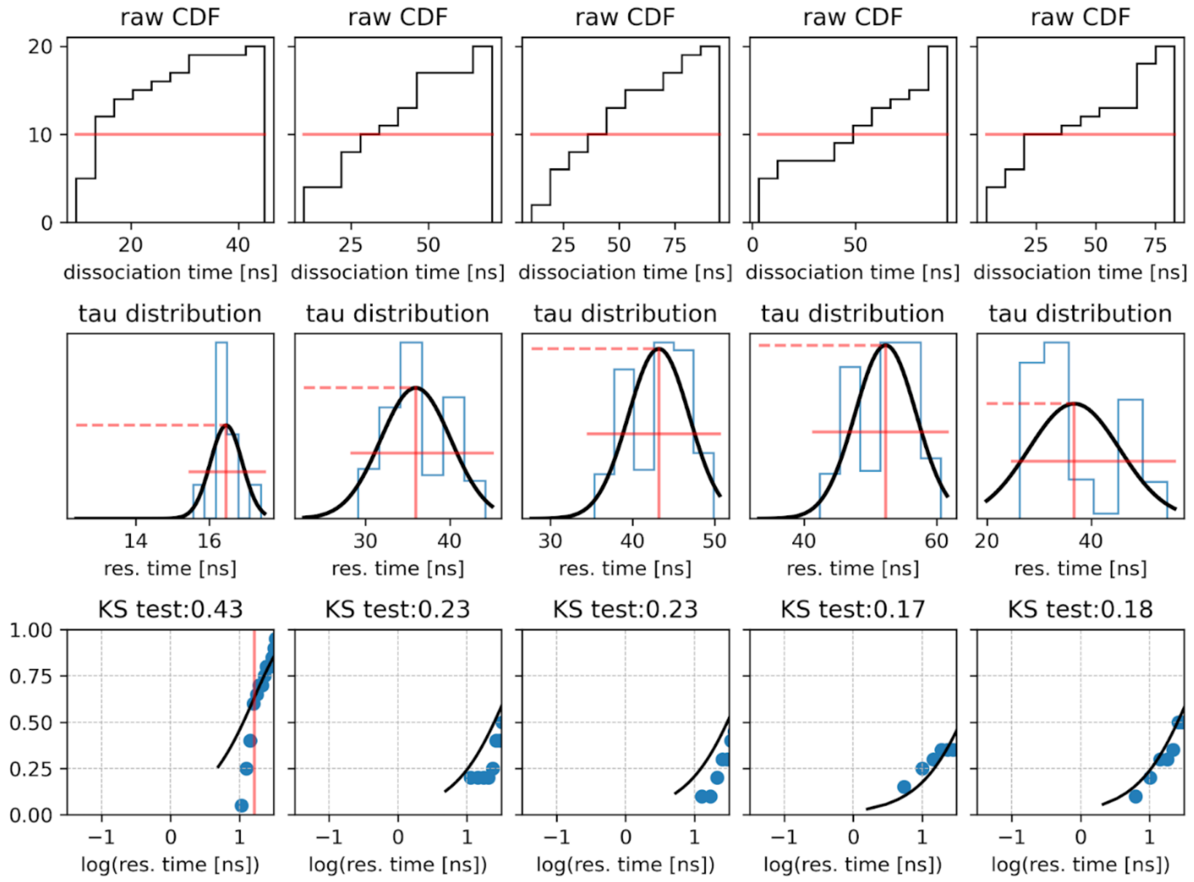

Figure S8. Distribution of residence times of ligand **1**. Top: cumulative distribution function (CDF) of residence times obtained from tauRAMD trajectories of ligand 1 in five replicas (20 trajectories per replica). Middle: distribution of residence times obtained from tauRAMD trajectories. Bottom: distribution of residence times obtained from tauRAMD trajectories in log scale, and results of Kolmogorov-Smirnov (KS) test.

Figure S9 - Residence times of ligand 2.

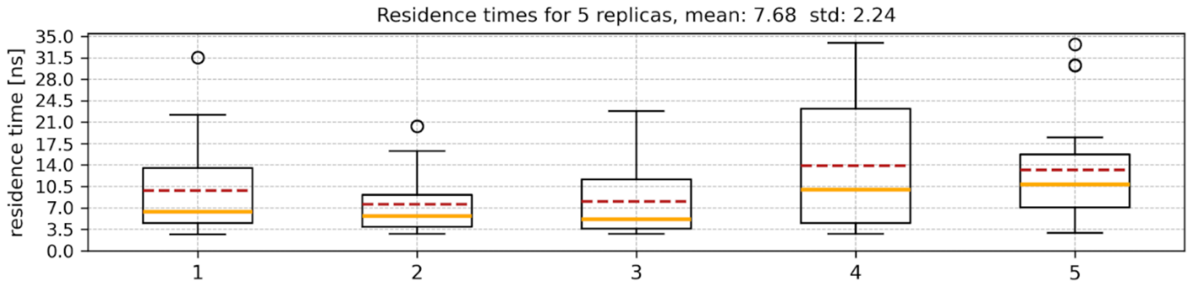

Figure S9. Residence times of ligand **2**. Box plots show the distribution of residence times obtained from tauRAMD trajectories of ligand **2** in five replicas (20 trajectories per replica). The yellow line displays the mean time, while the red dotted line shows the median of the residence times.

Figure S10 - Distribution of residence times of ligand 2.

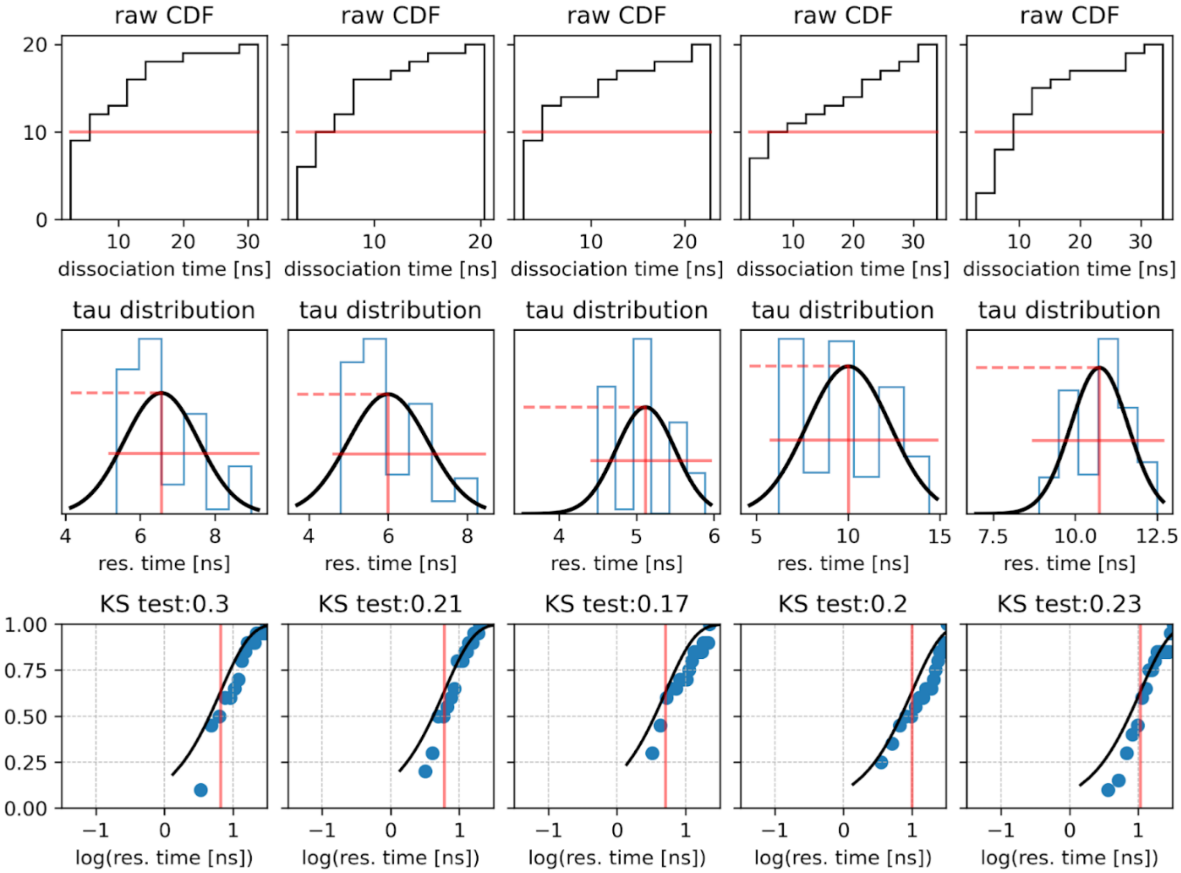

Figure S10. Distribution of residence times of ligand **2**. Top: cumulative distribution function (CDF) of residence times obtained from tauRAMD trajectories of ligand **2** in five replicas (20 trajectories per replica). Middle: distribution of residence times obtained from tauRAMD trajectories. Bottom: distribution of residence times obtained from tauRAMD trajectories in log scale, and results of Kolmogorov-Smirnov (KS) test.

Figure S11 - Residence times of ligand 3.

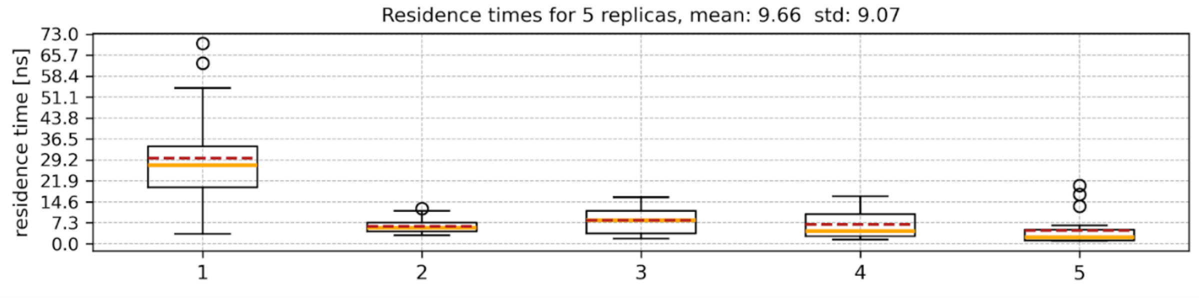

Figure S11. Residence times of ligand **3**. Box plots show the distribution of residence times obtained from tauRAMD trajectories of ligand **3** in five replicas (20 trajectories per replica). The yellow line displays the mean time, while the red dotted line shows the median of the residence times.

Figure S12 - Distribution of residence times of ligand 3.

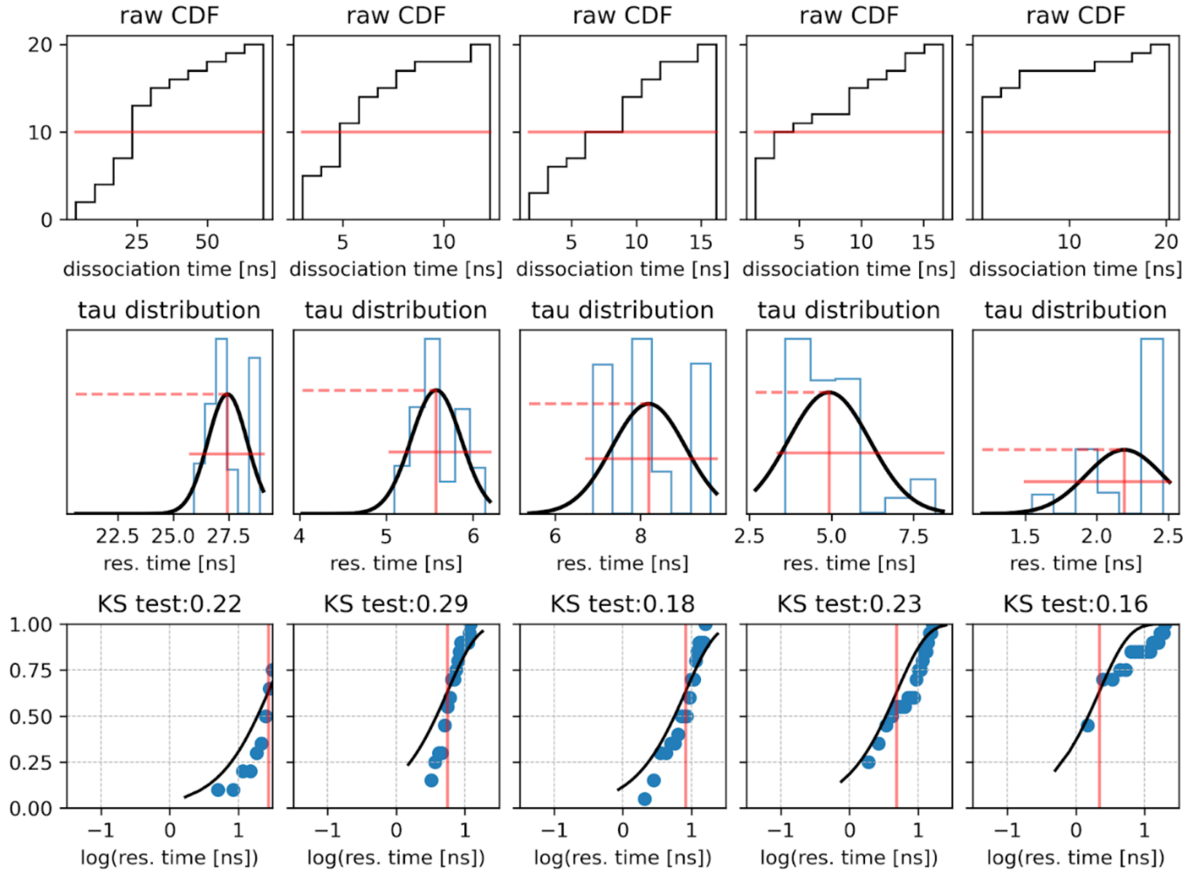

Figure S12. Distribution of residence times of ligand **3**. Top: cumulative distribution function (CDF) of residence times obtained from tauRAMD trajectories of ligand **3** in five replicas (20 trajectories per replica). Middle: distribution of residence times obtained from tauRAMD trajectories. Bottom: distribution of residence times obtained from tauRAMD trajectories in log scale, and results of Kolmogorov-Smirnov (KS) test.

Figure S13 - Residence times of ligand 4.

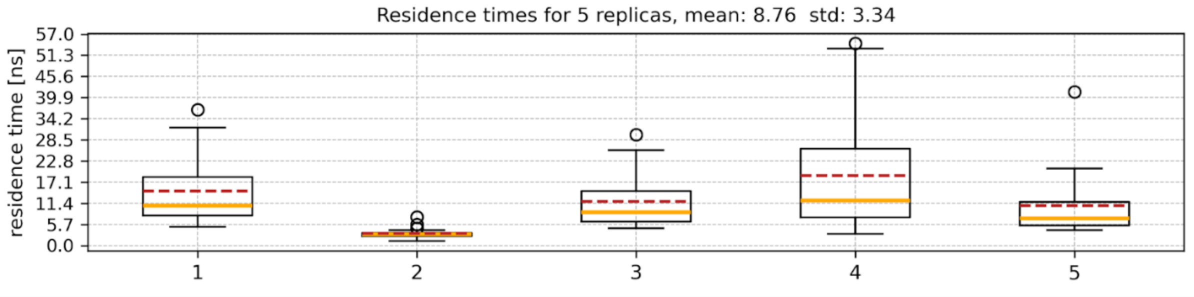

Figure S13. Residence times of ligand **4**. Box plots show the distribution of residence times obtained from tauRAMD trajectories of ligand **4** in five replicas (20 trajectories per replica). The yellow line displays the mean time, while the red dotted line shows the median of the residence times.

Figure S14 - Distribution of residence times of ligand 4.

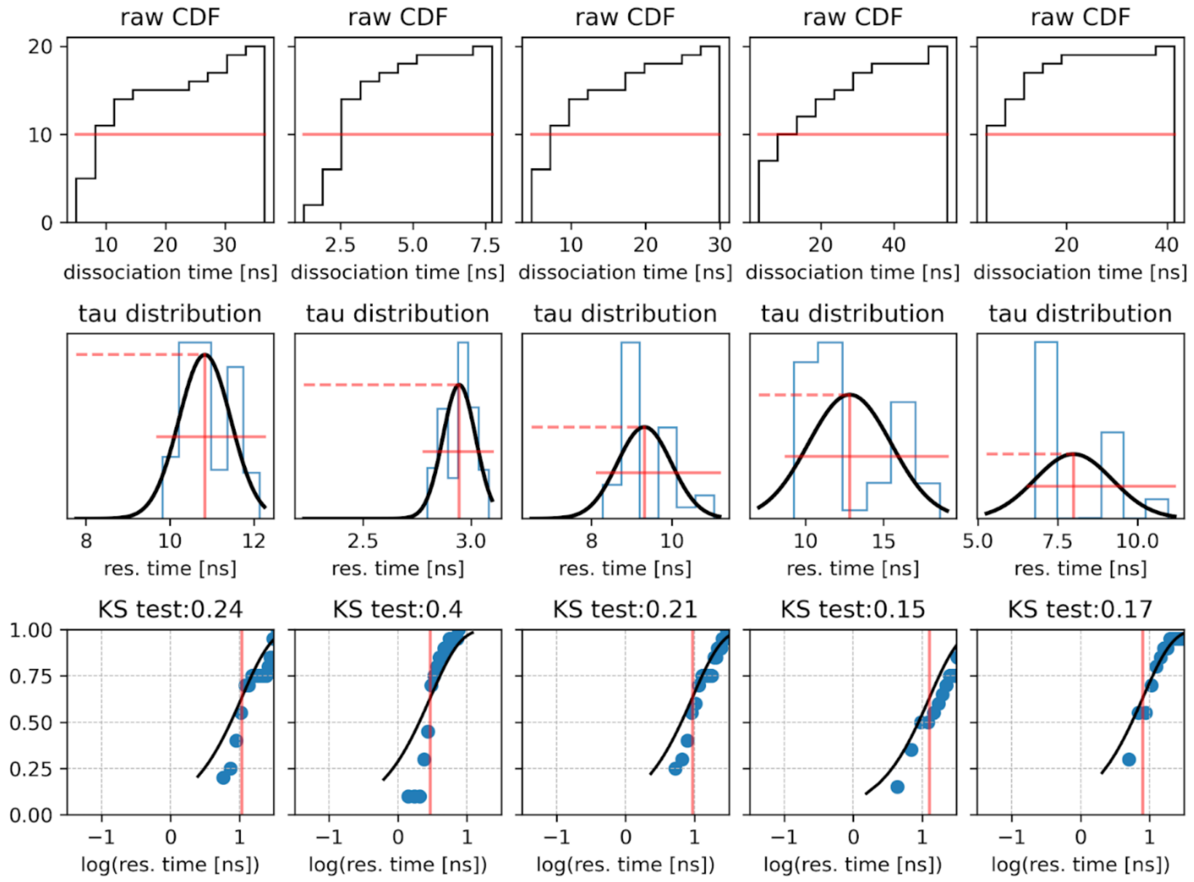

Figure S14. Distribution of residence times of ligand **4**. Top: cumulative distribution function (CDF) of residence times obtained from tauRAMD trajectories of ligand **4** in five replicas (20 trajectories per replica). Middle: distribution of residence times obtained from tauRAMD trajectories. Bottom: distribution of residence times obtained from tauRAMD trajectories in log scale, and results of Kolmogorov-Smirnov (KS) test.

Figure S15 - Residence times of ligand 5.

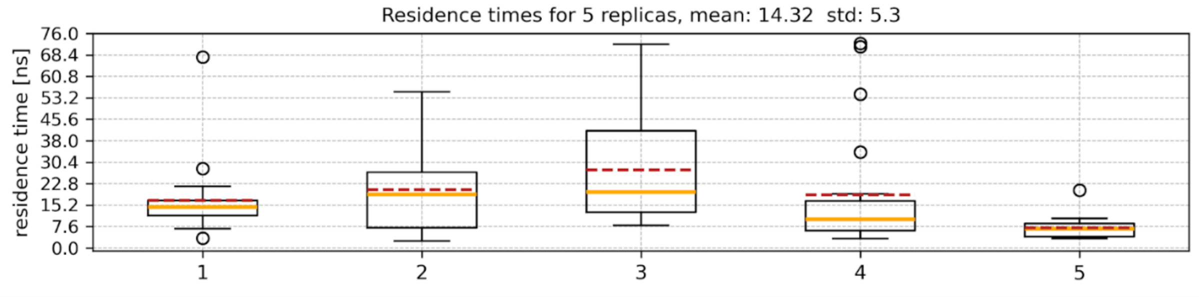

Figure S15. Residence times of ligand **5**. Box plots show the distribution of residence times obtained from tauRAMD trajectories of ligand **5** in five replicas (20 trajectories per replica). The yellow line displays the mean time, while the red dotted line shows the median of the residence times.

Figure S16 - Distribution of residence times of ligand 5.

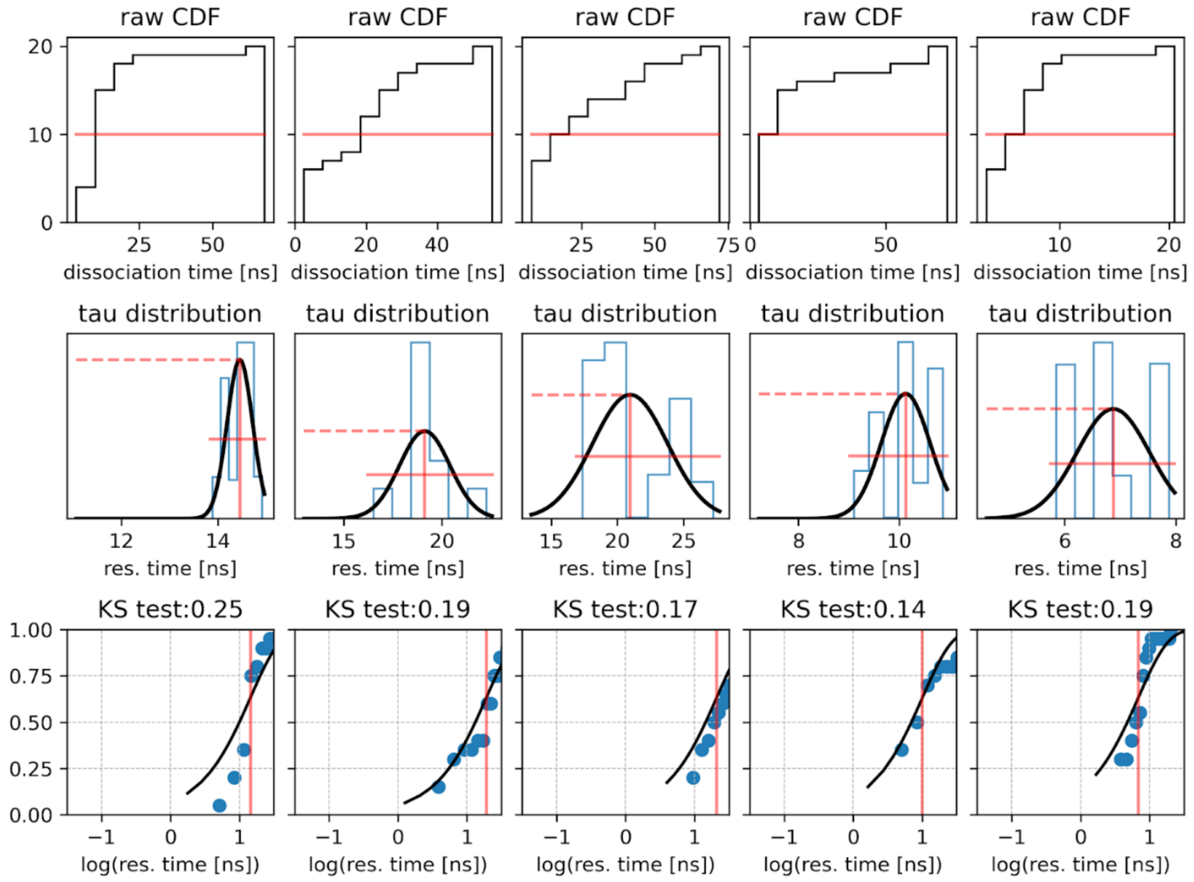

Figure S16. Distribution of residence times of ligand **5**. Top: cumulative distribution function (CDF) of residence times obtained from tauRAMD trajectories of ligand **5** in five replicas (20 trajectories per replica). Middle: distribution of residence times obtained from tauRAMD trajectories. Bottom: distribution of residence times obtained from tauRAMD trajectories in log scale, and results of Kolmogorov-Smirnov (KS) test.

Figure S17 - Residence times of ligand 6.

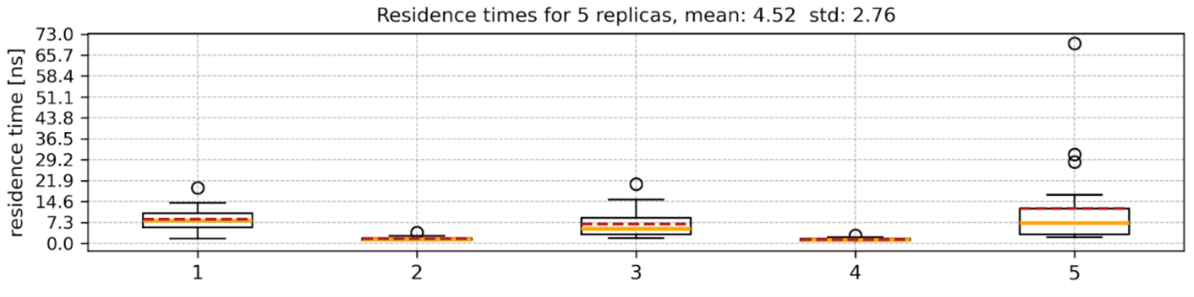

Figure S17. Residence times of ligand **6**. Box plots show the distribution of residence times obtained from tauRAMD trajectories of ligand **6** in five replicas (20 trajectories per replica). The yellow line displays the mean time, while the red dotted line shows the median of the residence times.

Figure S18 - Distribution of residence times of ligand 6.

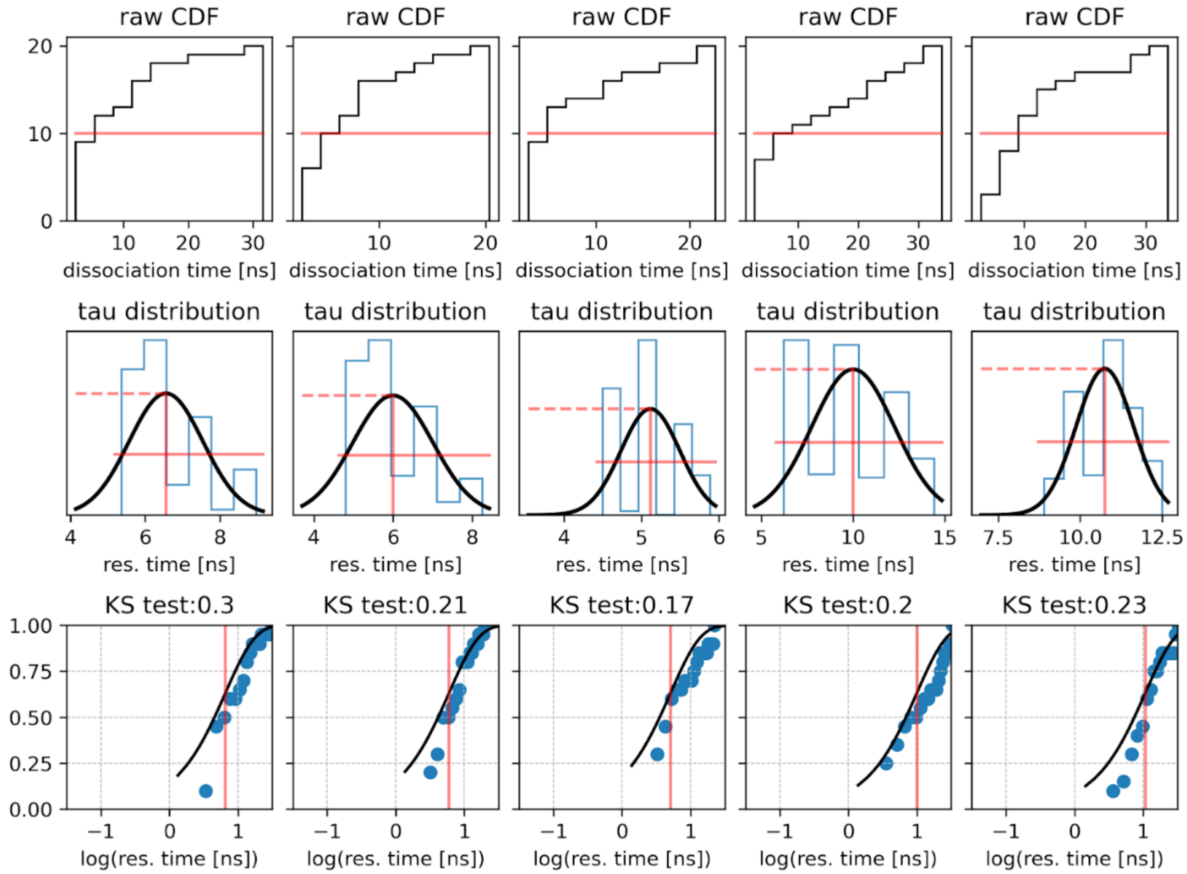

Figure S18. Distribution of residence times of ligand **6**. Top: cumulative distribution function (CDF) of residence times obtained from tauRAMD trajectories of ligand **6** in five replicas (20 trajectories per replica). Middle: distribution of residence times obtained from tauRAMD trajectories. Bottom: distribution of residence times obtained from tauRAMD trajectories in log scale, and results of Kolmogorov-Smirnov (KS) test.
